# Implementation of immunohistochemistry on frozen ear notch tissue samples in diagnosis of bovine viral diarrhea virus in persistently infected cattle

**DOI:** 10.1186/1751-0147-53-65

**Published:** 2011-12-05

**Authors:** Tomislav Bedeković, Nina Lemo, Ivana Lojkić, Ana Beck, Mirko Lojkić, Josip Madić

**Affiliations:** 1 Croatian Veterinary Institute, Savska cesta, 143, 10000 Zagreb, Croatia; 2 Faculty of Veterinary Medicine, University of Zagreb, Heinzelova 55, 10000 Zagreb, Croatia

**Keywords:** bovine viral diarrhea virus, diagnosis, ear notch frozen tissue, immunohistochemistry

## Abstract

**Background:**

Bovine viral diarrhea is a contagious disease of domestic and wild ruminants and one of the most economically important diseases in cattle. Bovine viral diarrhea virus belongs to the genus *Pestivirus*, within the family *Flaviviridae*. The identification and elimination of the persistently infected animals from herds is the initial step in the control and eradication programs. It is therefore necessary to have reliable methods for diagnosis of bovine viral diarrhea virus. One of those methods is immunohistochemistry. Immunohistochemistry on formalin fixed, paraffin embedded tissue is a routine technique in diagnosis of persistently infected cattle from ear notch tissue samples. However, such technique is inappropriate due to complicated tissue fixation process and it requires more days for preparation. On the contrary, immunohistochemistry on frozen tissue was usually applied on organs from dead animals. In this paper, for the first time, the imunohistochemistry on frozen ear notch tissue samples was described.

**Findings:**

Seventeen ear notch tissue samples were obtained during the period 2008-2009 from persistently infected cattle. Samples were fixed in liquid nitrogen and stored on -20°C until testing. Ear notch tissue samples from all persistently infected cattle showed positive results with good section quality and possibility to determinate type of infected cells.

**Conclusions:**

Although the number of samples was limited, this study indicated that immunohistochemistry on formalin fixed paraffin embedded tissue can be successfully replaced with immunohistochemistry on frozen ear notch tissue samples in diagnosis of persistently infected cattle.

## Findings

Bovine viral diarrhea is contagious disease of domestic and wild ruminants and one of the economically most important diseases in cattle. Bovine viral diarrhea virus (BVDV) belongs to the genus *Pestivirus*, within the family *Flaviviridae *[1]. Based on viral RNA sequence, there are at least 2 viral genotypes of BVDV that can be further divided into subgenotypes [2,3].

**Figure 1:**
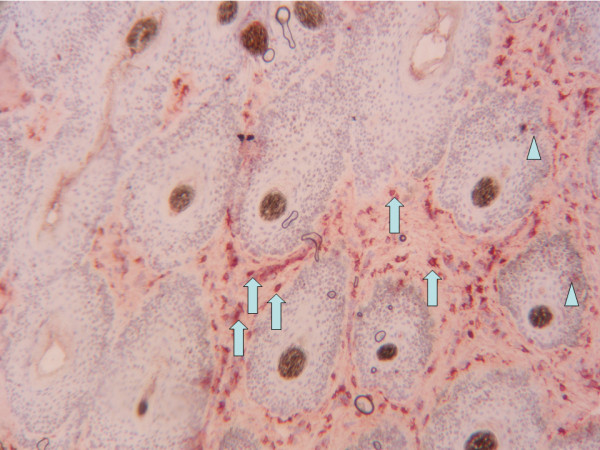
**Haired skin/pinna from a heifer persistently infected with bovine viral diarrhea virus (BVDV)**. Characteristic red cytoplasmic high staining of fibroblast and dendritic cells in interfolliculare space (arrow). Low keratinocytes staining in outer layers of some hair bulbs (head of arrow). Magnification 100 ×.

**Figure 2:**
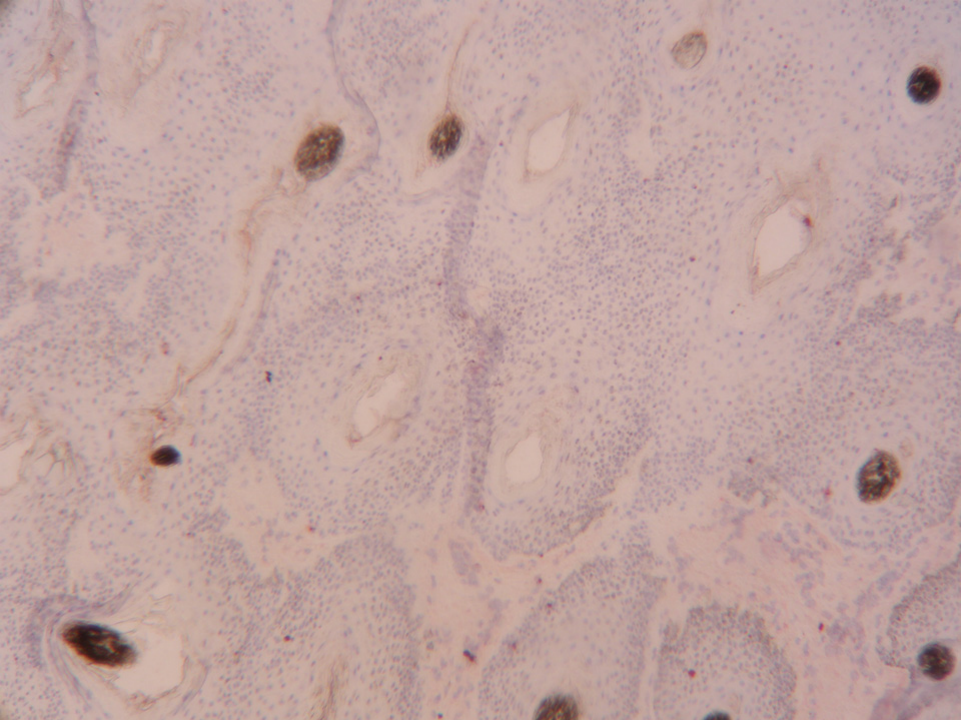
**Haired skin/pinna from a BVDV negative heifer**. Magnification 100 ×.

Furthermore, isolates of BVDV can be separated into non-cytopathogenic and cytopathogenic biotypes based on cytopathogenic effects observed in infected cell cultures [4,5]. Non-cytopathogenic strains have the possibility to cause persistent infection. Infection of heifers or cows with non-cytopathogenic biotype of BVDV in the first four months of pregnancy can lead to the birth of persistently infected (PI) calves [6]. PI animals act as a viral reservoir and are the main factor of the virus's continuation within herds [7]. To confirm the persistent infection, two samples should be taken within three to four week intervals. The identification and elimination of the PI animals from the herds is the initial step in the control and eradication programs. It is therefore necessary to have reliable methods for detection of PI animals. In diagnosis of BVDV, many different reliable methods are available. Virus or virus antigen can be detected in blood, sera, and organs from dead animals. Virus isolation (or its modification - the immunoperoxidase test) is still considered as a golden standard in diagnosis of BVDV. However, in cattle younger than six months, detection of the virus in sera samples can be adversely affected by the presence of specific antibodies [8]. Because of that, the reverse transcription polymerase chain reaction (RT-PCR) can be applied as a relative golden standard on sera samples [9] or ear notch tissue samples can be tested. Ear notch tissue samples can be tested with antigen immunoassay test (Ag ELISA), RT-PCR or immunohistochemistry (IHC). However, the distribution of viral antigen within tissue can be established only with IHC. IHC on formalin fixed, paraffin embedded tissue is a routine technique in diagnosis of PI cattle from ear notch tissue samples. However, such approach has many disadvantages due to complicated tissue fixation process and long preparation time. IHC on frozen tissue in diagnosis of BVDV is usually applied on organs from dead animals. For all of these reasons we described for the first time the IHC on frozen ear notch tissue samples from live animals. Ear notch tissue samples were collected during the period 2008-2009 from 17 positive heifers. This study was approved by ethics committee of Veterinary Faculty University of Zagreb; number: 251-61-01/139-11-72. Virus was confirmed by Ag ELISA and testing was repeated after four weeks. At the second sampling, two samples of ear notch tissue per animal were taken. A sample for the Ag ELISA and RT-PCR tests was stored at -20°C. The other sample was placed immediately into liquid nitrogen for 1 min and then stored at -20°C. To prevent contamination, samples were put in sterile cryo tubes and then placed in liquid nitrogen. Samples were transported to the laboratory on dry ice in a portable refrigerator. Delivery time to the laboratory was between 3 and 6 h. In the laboratory, the samples were stored at - 20°C until testing. The Ag ELISA for detection of viral antigen on ear notch tissue was performed using commercially available kit according to manufacturer's instructions (Herdchek BVDV Ag/Serum Plus, IDEXX, Liebefeld-Bern, Switzerland). Prior to the test, ear notch tissue samples 1 × 1 cm in size were placed in individual sterile tubes with 2 ml of provided buffer and refrigerated overnight at 4°C. All 17 samples showed positive result by Ag ELISA and were tested once again with RT-PCR in the same manner as described previously [10], targeting the 441-bp fragment of the Npro genome region. All 17 samples were also positive by RT-PCR. For IHC, ear notch tissue samples that were fixed in liquid nitrogen and stored at - 20°C were sectioned with scalpel in the shape of a trapeze, approximately 2 × 1 cm in size. Tissue sections were quickly transported in the cryostat (LEICA; CM1500S, Wetzlar, Germany) and fixed onto holders with the tissue freezing medium (LEICA, Nussloch, Germany). Tissue sections were cut at -25°C at 6 μm, mounted onto silane-coated slides (DAKO, Glostrup, Denmark), immersed in a solution of 80% acetone and dried overnight. Between each section, the blade was disinfected with 70% ethanol. Two samples were used for negative control; one, with confirmed negative result, and the second, a sample from PI cattle, where, during the IHC procedure, distilled water was used instead of specific monoclonal antibody. For staining and visualization an EnVision+Sistem-HRP commercial kit was used (DAKO, Glostrup, Denmark). Surface around each sample area was marked. The slides were placed in Tris-Buffered NaCl washing solution bath (DAKO, Glostrup, Denmark) bath during 10 sec. The excess fluid was then wiped around sample area with absorbent paper. To reduce nonspecific staining, sections were covered with the sera of rabbit origin (INVITROGEN, Carlsbad, USA) and held on room temperature in wet chamber for 10 min. Sera was discarded and slide was wiped around sample area. The completed sections were then covered with peroxidase blocking reagents provided in the same kit and slides were placed in wet chamber and held at room temperature (25°C). Slides were rinsed with washing solution by directing spray over the sample area and then placed shortly (30 sec) in washing solution bath. The excess fluid was wiped around sample area with absorbent paper. The completed sections were then covered with pool of four murine BVDV specific monoclonal antibodies (Monoclonal antibodies to pestiviruses, MAbMIX, BVD specific; VETERINARY LABORATORY AGANCY, Weibridge, UK) diluted 1:200 with antibody diluted reagent (DAKO, Glostrup, Denmark) and slides were placed in wet chamber and held in incubator at 37°C for 30 min. Slides were rinsed with washing solution by directing spray over sample area and placed shortly (30 sec) in washing solution bath. The excess fluid was wiped around sample area with absorbent paper. Sections were covered with anti-mouse antibodies provided in the kit and slides were placed again in in wet chamber and held in an incubator at 37°C for 30 min. Slides were washed again as described above. The excess fluid was wiped around sample area with absorbent paper. Sections were covered with substrate provided in the kit and slides were placed at room temperatures for 30 min. Slides were rinsed with washing solution by directing spray over sample area and placed shortly (30 sec) in washing solution bath. The excess fluid was wiped around sample area with absorbent paper. Slides were placed shortly (1 min) in contrast staining solution (DAKO, Glostrup, Denmark) and rinsed with distilled water. Each slide was placed shortly six times in 0.037 M ammonia solution and then slides were dried. Cover slides were fixed with mounting media (DAKO, Glostrup, Denmark) and slides were checked under microscope at 100 × magnification. Section was considered positive if specific red staining was observed in cytoplasm of cells. In all tested samples, positive results were obtained (Figure 1) and negative controls obtained negative results (Figure 2). In diagnosis of BVDV, IHC is popular technique because of the convenience of sample collection, and reliability [11]. Even Ag ELISA is quick and reliable method, in some cases false positive results can be obtained [12]. For that reason, it is necessary to confirm the obtained results. This can be achieved with RT-PCR or IHC. However, only with IHC it is possible to define the type of the infected cells and to determine the distribution of antigen within the tissue. This fact doesn't have the impact on the diagnosis of BVDV, but can be very useful in the study of virus pathogenesis. Because of the small prevalence of PI animals (up to 2%) and for the consequence a small number of samples, IHC is the more cost-effective method compared to RT-PCR. However, IHC on formalin fixed, paraffin embedded tissue requires more days for preparation (up to 5), and it is a multistep process prone to a technical error [11]. Another disadvantage is complicated tissue fixation process. Additionally, IHC on frozen ear notch tissue was quick and simple, and complete procedure was performed for maximum of 24 h. For the fixation of samples previously placed in cryo tubes, liquid nitrogen farm tanks can be used. After the fixation, samples should be stored at -20°C prior transportation to the laboratory. Also, transportation in portable refrigerator has no influence on the results and section quality. Two important facts should be bear on mind during the IHC procedure on frozen ear notch tissue samples. First, it is very important that samples were cut on cryostat with blade for bones because of ear tissue structure. Cutting with standard cryostat blade has not provided satisfactory sections quality. Second, very important part of the staining procedure is keeping the sections wet. To accomplish that, during the incubation steps, the slides should be placed in a wet chamber. Although the number of samples was limited, this study indicated that IHC on formalin fixated paraffin embedded tissue can be successfully replaced by IHC on frozen ear notch tissue samples in diagnosis of PI cattle.

## Competing interests

The authors declare that they have no competing interests.

## Authors' contributions

TB: Main initiator, designer and performer of the study and author of the manuscript; NL, IL, AB, ML, JM: Contributor to design, perform and revising the manuscript. All authors read and approved the final manuscript. Please see sample text in the instructions for authors.
